# Internet-delivered guided self-help Acceptance and Commitment Therapy for family carers of people with dementia (iACT4CARERS): a qualitative study of carer views and acceptability

**DOI:** 10.1080/17482631.2022.2066255

**Published:** 2022-04-17

**Authors:** Milena Contreras, Elien Van Hout, Morag Farquhar, Lance M McCracken, Rebecca L Gould, Michael Hornberger, Erica Richmond, Naoko Kishita

**Affiliations:** a School of Health Sciences, University of East Anglia, Norwich, UK; b Department of Psychology, Uppsala University, Sweden; c Division of Psychiatry, University College London, London, UK; d Norwich Medical School, University of East Anglia, Norwich, UK; e Older People’s Community Team, Norfolk and Suffolk NHS Foundation Trust, Norwich, UK

**Keywords:** Intervention acceptability, online therapy, informal caregivers, therapeutic relationship, self-compassion

## Abstract

**Purpose:**

To explore carers’ views and acceptability of internet-delivered, therapist-guided, self-help Acceptance and Commitment Therapy (ACT) for family carers of people with dementia (iACT4CARERS).

**Methods:**

A qualitative approach with semi-structured interviews was employed with family carers (N = 23) taking part in a feasibility study of iACT4CARERS. The interviews were audio-recorded, transcribed and analysed using thematic analysis.

**Results:**

Four overarching themes were identified: 1) usefulness and relevance of the content of the sessions, 2) sense of connectedness, 3) the impact of the intervention on participants and 4) acceptability of the online delivery. Positive carer experiences and intervention acceptability were facilitated by learning helpful ACT skills, perceiving the content of the session as relatable to the carers’ needs, feeling connected to other carers and the therapist during the intervention, noticing the benefits of the intervention and the user-friendliness of the online platform. Recommendations for a full-scale trial were identified, such as the inclusion of some “face-to-face” interactions (e.g., via video call) between carers and therapists to facilitate a bidirectional interaction and the provision of an additional aide-memoire to improve the learning experiences.

**Conclusion:**

Overall, the intervention was acceptable to the family carers. The proposed recommendations should be considered in a full-scale trial.

## Introduction

There is considerable evidence that family carers of people with dementia are at a higher risk of developing anxiety and depression (Collins & Kishita, 2020; Kaddour & Kishita, 2020). Poor mental health is often associated with long-term physical health problems among dementia carers (Pinquart & Sörensen, 2007). Subsequently, this can lead to negative consequences for the care recipient, such as a reduction in their quality of life, increased use of healthcare services and institutionalization (Eska et al., 2013; Guterman et al., 2019; Martyr et al., 2018). Despite this, many carers are not able to access timely psychological support due to various barriers, such as reduced mobility, geographical location, lack of respite care, a shortage of skilled therapists leading to long waiting lists, and difficulties in prioritizing their own health needs (Green et al., 2012; Lappalainen et al., 2021; Tatangelo et al., 2018).

It is estimated that one million people in the UK will have dementia by 2025, increasing to two million by 2050 (Prince et al., 2014). As a consequence, the annual economic cost of dementia in the UK is expected to double from £26.3 billion to £55 billion by 2040 (Prince et al., 2014). It is estimated that over 60% of people with dementia are living in the community in the UK and around 87% of them receive help from their family members in their daily life (Prince et al., 2014; Alzheimer’s Society, 2017). Therefore, there is an urgent need to address the challenges of treatment accessibility and scalability in family carers. One way to do this is to design a service that can be delivered remotely at a time chosen by the carer and that requires only minimal support from healthcare professionals. Evidence-based, online, self-help interventions may fit the need in this population.

Acceptance and Commitment Therapy (ACT) is an evidence-based psychotherapy, which aims to promote psychological flexibility—the ability to step back from difficult thoughts and approach or allow painful emotions, focusing on the present, connecting with what is happening in the moment, clarifying and acting on what is most important to do and building larger patterns of effective values-based behaviour (Hayes et al., 2013). Recent systematic reviews have demonstrated that in-person ACT is effective in improving outcomes such as mood and quality of life in various populations including people with depression, anxiety, chronic pain, and somatic health problems (A-Tjak et al., 2015; Gloster et al., 2020; Hann & McCracken, 2014).

Recent randomized controlled trials (RCTs) of in-person ACT for family carers of people with dementia also demonstrated that face-to-face ACT delivered by qualified psychologists is effective in treating anxiety and depression in family carers of people with dementia (Losada et al., 2015; Márquez-González et al., 2020). Furthermore, recent systematic reviews on internet-delivered ACT suggest that ACT skills can be learnt online and may improve the psychological well-being of nonclinical (e.g., students) and somatic (e.g., people with chronic pain) populations (Brown et al., 2016; Thompson et al., 2021; Trindade et al., 2021), although research on internet-delivered ACT for family carers of people with dementia remains scarce.

Following these recent developments in ACT, a feasibility study was conducted aiming to (a) develop an internet-delivered, guided, self-help ACT intervention for family carers of people with dementia; (b) explore whether this new intervention is acceptable among this population; and (c) test whether it is feasible to deliver this intervention within the formal healthcare (i.e., NHS) services in the UK (Kishita et al., 2021).

As part of the earlier completed feasibility study, this qualitative interview study was conducted to evaluate the acceptability of internet-delivered, guided, self-help ACT among family carers of people with dementia. Qualitative research within a feasibility study is critical for addressing issues concerning acceptability, implementation and practicality of interventions. Given the often subtle and complex structures of these issues, qualitative analysis can offer in-depth insights on which aspects of the intervention should be improved: a topic that may not be fully captured in the quantitative findings (O’Cathain et al., 2015).

Following the theoretical framework of acceptability of interventions (TFA; Sekhon et al., 2017), individual interviews explored attitudes towards the intervention, and perceived effectiveness by addressing the following research questions: (a) What is perceived by family carers as helpful or unhelpful regarding the content and delivery method of the intervention?; (b) What benefits and harms did they experience from the intervention?; (c) From a carer perspective, how could the intervention be refined to make it more acceptable in a full-scale trial?

## Materials and methods

### Study design

This qualitative study used semi-structured interviews to explore the views on and acceptability of internet-delivered, guided, self-help ACT among family carers of people with dementia who participated in a feasibility study of the intervention (ISRCTN trial registration number: 18956412). The consolidated criteria for reporting qualitative research (COREQ) checklist (Tong et al., 2007) is shown in the online supplemental material 1. Full ethical approval was obtained from the NHS London—Queen Square Research Ethics Committee (20/LO/0025). All participants provided written consent via post or electronically as part of the main feasibility study.

### Intervention

After confirming eligibility, participants (i.e., family carers) were given access to the online programme. In brief, the intervention consisted of eight online sessions in which different interactive exercises illustrating ACT skills tailored for family carers were presented using audio, video and text-based materials. The contents of each session are described in Table I. At the end of each session, a reflective section was presented in which participants were encouraged to reflect on exercises and leave comments for the online therapist. This was restricted to a single comment from the participant and a single response from the therapist for each session and the programme did not offer further bidirectional interaction. The minimally trained novice therapists (e.g., assistant psychologists) were recruited from NHS services. Individually personalized feedback was provided by the online therapists to support participants’ self-learning. Each session had a gap of seven days before the next session became available. Participants were encouraged to complete all sessions within 12 weeks and were informed that access to the intervention would cease after this period.

**Table I.** Content of each session of the iACT4CARERS intervention

	ACT process	Content of the session (learning objectives); by the end of the session, participants will be able to:
Session 1	Introduction to ACT Creative hopelessness Openness	Describe the ACT model and the main goal of this therapy See the potential adverse impact of common control strategies (denial, avoidance and suppression of feelings) Engage with the present moment awareness exercise
Session 2	Values Introduction to committed actions	See the differences between values and goals Identify the important areas of their life Be aware of a lack engagement with some areas of their life (e.g., self-care) Identify the smallest step they are willing to take that would move them towards valued directions
Session 3	Overcoming external barriers	See the differences between common external barriers and internal barriers experienced by carers of people with dementia Come up with ideas to overcome their own external barriers using selective optimization and compensation in order to take committed actions Identifying the smallest step they are willing to take that would move them towards valued directions
Session 4	Overcoming internal barriers (cognitive fusion)	See the potential adverse impact of common control strategies (controlling thoughts) Engage with various defusion exercises Identifying the smallest step they are willing to take that would move them towards valued directions
Session 5	Overcoming internal barriers (awareness and openness)	See what it means by present moment awareness Engage with various present moment awareness exercises Identifying the smallest step they are willing to take that would move them towards valued directions
Session 6	Self-compassion	See the potential adverse impact of repetitive self-critical judgements Engage with a self-compassion exercise Identifying the smallest step they are willing to take that would move them towards valued directions
Session 7	Building a pattern of effective action	Reconnect with their values Describe a pattern of effective action that reflect their values Identify helpful steps to take when emotional distress is too painful Identifying the smallest step they are willing to take that would move them towards valued directions
Session 8	Summary of ACT Preparing for the future	Summarize all ACT skills learned Identify some future challenges and see how some of ACT skills may be useful to address them

Note. ACT = Acceptance and Commitment Therapy

In addition, participants had the option to attend three online peer-support groups while completing the online intervention. This option was only available if three or more participants signed up for groups within three weeks from the date of the screening session. If not, participants were asked to start the online intervention without this option. Peer-support groups were conducted as video calls via Microsoft Teams and were facilitated by one of the online therapists. There were no explicitly planned therapeutic elements to these peer support groups, and the sessions were led by the participants. More information about the intervention is available in the report on the feasibility study summarizing the quantitative results of the study (Kishita et al., 2021).

### Participant selection

Participants were recruited through clinician referrals from three National Health Service (NHS) services in England. The study was also advertised in local newspapers and on Join Dementia Research (a national online recruitment tool). Participants who had previously participated in other approved non-interventional dementia studies led by the same study team, and who had consented to be contacted about future studies, were also invited. Inclusion criteria for the feasibility study were: (a) being aged 18 and over, (b) being an unpaid carer for a relative with a clinical diagnosis of dementia, (c) identifying oneself as the primary carer, and (d) presenting with mild-to-moderate depression or anxiety as indicated by a score of 6–15 on the Generalized Anxiety Disorder-7 (Spitzer et al., 2006) or 6–15 on the Patient Health Questionnaire-9 (Kroenke et al., 2001). Participants were excluded if they were currently (a) receiving psychological treatment, (b) suffering from severe and poorly controlled psychiatric disorder or other conditions that may impair treatment engagement (e.g., cognitive impairment) or (c) had no access to the internet.

All feasibility study participants were invited to participate in a semi-structured interview after they, either, completed all eight online sessions or they lost their access to the online programme after 12 weeks (whichever came first). Thirty-three participants were enrolled in the intervention phase, of which nine participants were lost to the follow-up. Of the remaining 24 participants, 23 agreed to take part in the follow-up interview. One participant declined to take part due to increased caring responsibilities.

### Data collection

The feasibility study (intervention phase and follow-up interviews) was conducted during the COVID-19 pandemic, thus all semi-structured interviews were conducted via telephone or Microsoft Teams video call, depending on career preference. The interviews lasted less than an hour, and no one was present apart from the interviewer and participant during the session.

All interviews were conducted by two researchers (EVH, MC) except for four interviews, which were conducted by a research assistant (female) under the close supervision of NK (psychologist, PhD). EVH (female nurse, MSc) and MC (female psychologist, MSc) were experienced in conducting interviews. All interviewers conducted the baseline assessment with participants as part of the feasibility study. Thus, interviewers had a relationship established with participants prior to the commencement of follow-up interviews. The purpose of conducting this qualitative study was clearly explained at the beginning of the interview. To ensure consistency, an interview guide with open-ended questions and prompts to help explore participants’ views and acceptability of the intervention, developed based on consensus among the research team members, was used (see supplemental material 2). Participants did not receive these questions beforehand. Digital audio recordings were transcribed verbatim. All transcripts were anonymized and checked for accuracy.

### Analysis

This study included a thematic analysis to explore the most common thoughts about the intervention and to understand if the participants found the intervention acceptable or not. The “codebook” approach was followed using a structured coding framework for developing and documenting the analysis while themes were developed and refined through inductive data engagement and the analytic process (Braun & Clarke, 2021). The analysis was led by EVH and MC with co-analysis by NK. The coding process consisted of two steps: (1) researchers independently coded six interviews, comparisons were made, and a consensus on the codes was obtained, (2) the remaining transcripts were then coded by EVH guided by a list of codes created during the first step. Codes were added to the list within collaboration and by mutual agreement of two researchers (EVH, NK). NVivo V12 was used to assist the researcher in the organization and coding of the data.

Once all the transcripts were coded, potential themes and sub-themes were identified and refined based on the agreement between three researchers (EVH, MC, NK). To avoid any potential bias in the data analysis due to the relationship between researchers and participants, three public advisory members with experience of supporting a family member living with dementia were invited to the further data analysis process. This process involved the presentation of the potential themes and sub-themes, followed by an exhaustive discussion. Any controversy or uncertainty that arose during the meeting were resolved in mutual agreement.

## Results

**Figure 1.**
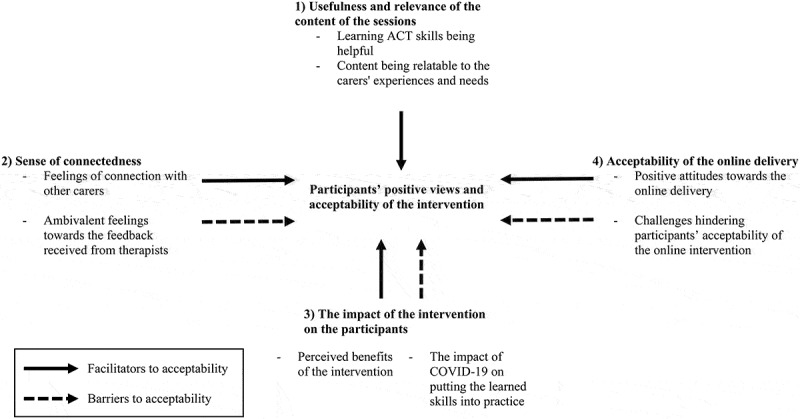
Overview of overarching themes and sub-themes.

Twenty-three participants were interviewed between November 2020 and April 2021; their characteristics are summarized in Table 2. Four overarching themes of the family carers’ views on iACT4CARERS were identified: (1) *Usefulness and relevance of the content of the sessions*, (2) *Sense of connectedness*, (3) *The impact of the intervention on participants*, and (4) *Acceptability of the online delivery*. An overview of the overarching themes and sub-themes is provided in Figure 1anda narrative with supporting illustrative quotes are provided below.

**Table 2.** Characteristics of participants (N = 23)

Carer age (Mean and SD)	62.91 (10.73)
Carer gender	
Female	20 (87%)
Male	3 (13%)
Relationship with the care recipient	
Spouse	12 (52%)
Child	10 (44%)
Sibling	1 (4%)
Hours of caring per week	
0–2 hours per week	2 (9%)
3–10 hours per week	3 (13%)
11–20 hours per week	2 (9%)
21–40 hours per week	2 (9%)
41–80 hours per week	5 (20%)
81 or more hours per week	9 (40%)
Care recipient’s dementia diagnosis	
Alzheimer’s disease	6 (26%)
Vascular dementia	3 (13%)
Dementia with Lewy bodies	1 (4%)
Frontotemporal dementia	1 (4%)
Mixed dementia	9 (40%)
Other	3 (13%)
Number of online sessions completed (out of 8)	
8 sessions	18 (78%)
7 sessions	4 (18%)
4 sessions	1 (4%)
Optional peer support group participation	
Attended 0 peer support groups	16/23
Attended 1 peer support groups Attended 2 peer support groups	7/23 7/23
Attended 3 peer support groups	6/23

### Usefulness and relevance of the content of the sessions

Two sub-themes were identified: “Learning ACT skills being helpful” and “Content being relatable to the carers” experiences and needs’.

#### Learning ACT skills being helpful

The family carers taking part in the study expressed that the ACT skills learned throughout the intervention were helpful for improving their experiences of looking after a family member living with dementia. Among the different ACT psychological-flexibility-promoting techniques introduced in the intervention, the participants particularly valued learning to notice and allow painful emotions while focusing on the present and connecting with what is happening in the moment. These skills enabled carers to step back from their worries or feelings of guilt and to appreciate the time spent with the care recipient. However, above all, the most valued exercise among the majority of participants, but also equally highlighted as one of the most emotionally difficult exercises, was an audio-guided, self-compassion exercise.

“I really practised keeping myself in the present, so that I could really enjoy her company. Because that’s something I know I tend to do, my mind tends to start rushing forward to either what I’ve got to do later that day or, you know, something that’s happened yesterday.” (Participant 11, wife).

“I do think it helped me to sort of move outside of myself and look at myself from the outside. Looking like I was a friend and thinking to myself. If it was my friends, ‘what would I be saying to them?’ So, I found that quite helpful, to sort of say these things to myself. You are doing a hard, quite difficult job and it is okay to sort of feel sometimes angry or resent or tired. You know, to not feel bad about yourself. Keep a sort of compassion, I found that very useful.” (Participant 04, daughter).

#### Content being relatable to the carers’ experiences and needs

Another aspect that appeared to facilitate acceptability of the intervention was the simplicity and relevance of exercises and metaphors used to illustrate ACT techniques. The participants felt that the exercises and metaphors were simple and easy to understand and to remember, particularly when they were struggling with their caregiving tasks; despite this simplicity they were also still effective. Moreover, the imaginary carer “Mrs Stewart”, a lady looking after her husband with dementia, used throughout the intervention to demonstrate when and how ACT techniques could be implemented, was also highly appreciated by the participants. The participants expressed that they were experiencing similar obstacles to the example of “Mrs Stewart” and that seeing Mrs Stewart overcoming challenges using ACT skills was inspiring.

“ … you used lots of metaphors to help you remember, because when you’re caring for the person that you’re caring for, when it’s in the heat of the moment and when you’re on the frontline, sometimes it’s hard to remember things. So using the metaphors like the garden and the tree in the centre of the garden, that really helped me in the heat of the moment to remember what I learnt and to recap on it.” (Participant 16, daughter).

### Sense of connectedness

Two sub-themes were identified: “Feelings of connection with other carers” and “Ambivalent feelings towards the interactions with therapists”.

#### Feelings of connection with other carers

Even though the carers were completing the intervention on their own and, in most cases, without any interaction with other participants, many of them reported feeling somehow connected with other carers. Learning that their thoughts, feelings and difficulties were normal and common among dementia carers, made the participants feel more accepting of themselves and less lonely. This feeling of connection with other carers was also increased by knowing that other participants were completing the intervention.

“I was quite amazed about how common the things that I think and feel … because I thought it was just me. When I was reading things, when people are looking after their relatives and everything, I thought I was just the one who got so worried and frustrated. (…) Basically, it’s taught me that everything I think, and feel is quite normal.” (Participant 20, daughter).

“And knowing that there were people all over the country experiencing this and dealing with this and listening to the same programme that I was listening to, it did make me feel part of a community. (…) although I didn’t attend any of the support groups, I did get the really strong feeling that I wasn’t alone. Because I really felt that giving other examples and just knowing that this programme was there for lots and lots of people because they wouldn’t just do it for me, you know what I mean, and I did feel less alone.” (Participant 09, sister).

For those participants that attended the peer support groups this sense of connectedness was even more evident. These carers felt that taking part in the peer support groups allowed them to meet and talk to people that were going through a similar situation and that could truly relate to their struggles. Moreover, the participants felt that it was helpful to learn from other carers’ experience with the intervention and with their role in looking after a person with dementia. This additional opportunity appeared to facilitate acceptability of the intervention.

“I think it’s just nice to connect with other people, and maybe not in exactly the same situation as you but are facing very similar things in their lives. (…) because they all know what it’s like, you know, when you’re living alongside somebody with dementia. They all understand what it’s like, whereas friends don’t always understand, and family don’t always understand. They don’t see the whole.” (Participant 11, wife).

#### Ambivalent feelings towards the interactions with therapists

Another important element of the intervention, which enabled the sense of connectedness among the participants, was the interaction they had with their therapist. Most of the participants expressed that the feedback received from their therapist was encouraging and helped them continue with the intervention. One of the most valued aspects of feedback reported was feelings of being listened to by someone and reassurance from their therapist that the way they were feeling was normal among carers.

“What worked a bit when she came back to me that other people feel just like I do. That really helped. It helped me to feel like I was not alone really in this. Maybe. I think that was just really good, you know. Knowing that someone is listening to me. I listen to my parents all the time. And someone was actually listening to me and said, ‘okay this is normal how you feel’. That was good.” (Participant 05, daughter).

However, some participants felt that the interactions with their therapist were not very helpful as they perceived that the therapists’ feedback was not personalized enough and, thus, it did not resonate with them. Further difficulty in the interactions with the therapists were reported. These included the delay in receiving feedback from their therapist (i.e., no live interaction) and the fact that the text-based feedback was explicitly limited to a single exchange after each session (i.e., no bidirectional interaction). The carers suggested including some “face-to-face” interaction with the therapists to facilitate a bidirectional interaction, which could improve the acceptability of the intervention.

“In the early stages, I wasn’t writing very much because, I suppose, I just didn’t, I didn’t feel that I had learnt an awful lot then, and I didn’t think, there wasn’t a lot to say, and so her responses were fairly bland, you know, but then as I wrote more as I went through it and wrote more, the responses were more helpful.” (Participant 03, wife).

“I found the feedback fairly helpful but the problem with not being able to have a conversation with somebody is that you can’t give feedback straightaway (…). I think the feedback, that’ll be useful to have that maybe face to face. It’s just to have some kind of two-way interaction.” (Participant 07, daughter).

### The impact of the intervention on participants

Two sub-themes were identified: “Perceived benefits of the intervention” and “The impact of COVID-19 on putting the learned skills into practice”

#### Perceived benefits of the intervention

There was clear evidence from the participants’ point of view that they benefitted from the intervention and several aspects of improvement in their life were reported. One of the commonly reported perceived benefits was increased awareness of their own needs. Many carers expressed that the intervention helped them to understand the importance of self-care and to prioritize their own needs rather than only focusing on the needs of their care recipient. Moreover, the intervention allowed the carers to ask for help from other people when needed without feeling too guilty for doing so. One of the most reported benefits of the intervention was increased value-based activities (e.g., doing more exercises to improve their own health). The intervention helped the carers to identify what truly mattered to them beyond caring for the person with dementia and to take small steps that would bring them closer to their values.

“It was very much the reflecting on what your particular attitude was to the person you’re caring for, and, and to open your mind to make sure that you were looking after your own wellbeing, as well as looking after the person that you’re caring for. (…) it reinforced the strong message that I should put in more effort to look after myself, rather than dedicating all my time to my wife who is the person in need of care … ” (Participant 01, husband).

“I’ve definitely asked for help more from my family. I wasn’t asking for help because they’ve got such a lot on themselves, but I thought I must let them know that I needed help, even if they couldn’t give me any.” (Participant 13, wife).

#### The impact of COVID-19 on putting the learned skills into practice

Despite the aforementioned benefits, many participants expressed that the COVID-19 pandemic hindered the opportunity of putting the learned skills into practice. Most of the participants completed the intervention when the UK was under public health restrictions (i.e., lockdown) and, therefore, the participants felt that they were not able to engage in activities they really wanted to do (e.g., meet friends). The intervention encouraged participants to take small steps that are feasible but still reflected their values, however, the content was not tailored enough for challenges related to the COVID-19 pandemic. For this reason, some participants felt that they would have gained more from the intervention if they had completed it on another occasion.

“(…) because of lockdown and being unable to carry through what I’d learnt during the sessions, you know, I wasn’t able to physically carry it through. I could do things like decide what I wanted to do and make phone calls, but I couldn’t do things like meet up with people. So that was difficult so it’s nothing to do with the programme, it was the time thing, but you know what, COVID doesn’t know when is a good time.” (Participant 07, daughter).

“I think I would have gained more from it [the intervention] if I was actually able to put some aims down and was able to actually act on more things than I was able to do during the course of the programme.” (Participant 12, wife).

### Acceptability of the online delivery

Two sub-themes were identified: “Positive attitudes towards the online delivery” and “Challenges hindering participants” acceptability of the online intervention’

#### Positive attitudes towards the online delivery

There was clear evidence that the delivery method of the intervention (i.e., internet-delivered) was acceptable to the participants. Many carers expressed that the online mode of delivery gave them more flexibility to complete the sessions and that the time and effort required to complete the intervention was perceived as not demanding. The combination of these factors appeared to facilitate acceptability of the mode of delivery, considering the constraints of being a carer. The participants also found that the online platform in which the intervention was delivered was easy to access and navigate, which was another aspect that increased the acceptability of the delivery method.

“I really liked the fact that it was online, because when you’re caring for someone, it’s hard to be in a particular place for a particular time, because you never know what’s going to happen (…). So, I just thought it was one less pressure for me, because I knew that I was in control of it.” (Participant 16, daughter).

“I didn’t have any problems logging in or anything like that. I managed to … if I wanted to go back and look at something, I managed to find it. I’m not very computer-savvy, so if I can do it, it will be okay.” (Participant 10, wife).

#### Challenges hindering participants’ acceptability of the online intervention

The carers highlighted some challenges they experienced in relation to the online delivery that perhaps could be improved in a full-scale trial. The participants were able to revisit all audio- and video-recorded exercises and metaphors from previous sessions throughout the intervention, however some felt that being able to only revisit these materials was not enough. They expressed that they wished they had the opportunity to revisit the whole session they completed so that they could check the responses they had made to questionnaires within the session (e.g., a questionnaire on their values). Participants also expressed that having to wait one week for the next session to become available was somewhat restrictive. Since the participants had a set time frame (i.e., 12 weeks) to complete all eight sessions they wished they had more flexibility on when to start the next session.

“So, sometimes I would have liked to have gone back over my answers to some of the sessions to be reminded of what I actually said or wrote because sometimes I’d be asked a question about something I might have identified earlier on and I’d be thinking do you know what, I really can’t remember what I identified. So, yes, I would have liked to have sort of been able to go back over things.” (Participant 07, daughter).

“One of the things that could be changed, I think, was once you’ve done one session, you have to wait seven days for your next session to be available, and unfortunately because I missed the two weeks with things that were going on in life, I was hoping that I would be able to catch up but not realising that I would have to wait seven days. (…) if there is a cut off time for the end of the course and people are behind, there should be that facility where they can catch up at their own pace.” (Participant 18, son).

## Discussion

This study aimed to understand the views and acceptability of internet-delivered, guided, self-help ACT among family carers of people with dementia. Four overarching themes were identified in the semi-structured interviews with carers: usefulness and relevance of the content of the sessions, sense of connectedness, the impact of the intervention on participants and acceptability of the online delivery.

The findings demonstrated that learning ACT skills that promoted psychological flexibility was helpful for the carers, and the relevance of the content and the usefulness of these techniques appeared to facilitate acceptability of the intervention. Common adverse psychological processes among family carers of people with dementia, include self-criticism, avoidance of positive emotional experiences (e.g., avoiding feelings of enjoyment from pleasurable activities) and feelings of guilt (Márquez-González et al., 2014, 2010). Previous studies demonstrated that self-compassion techniques can be effective for reducing such self-criticism and feelings of guilt and for improving acceptance of difficult emotions and thoughts among family carers (Gallego-Alberto et al., 2021; Han et al., 2021; Lloyd et al., 2019; Márquez-González et al., 2014). This may support why self-compassion was particularly appreciated by most of our participants. Moreover, a recent pilot study offering online ACT session to carers of people with dementia via videoconference (Han et al., 2021) demonstrated that the participants felt that being more self-compassionate helped them to reduce self-criticism, feelings of guilt and to start taking care of themselves following the intervention, which consistent with our findings.

There was clear evidence that participants perceived themselves as having benefitted from the intervention. Benefits, such as being able to allow oneself to ask for help from other people, increased awareness of own needs and increased engagement in value-based activities were also reported in a recent study where dementia carers completed an online ACT intervention (Han et al., 2021). There is considerable evidence supporting that the perceived benefits of interventions are linked with greater acceptability of the intervention (Berry et al., 2016; Morrison et al., 2014). This also appeared to facilitate acceptability in the current study.

The findings of this qualitative study also highlighted that, despite the virtual context in which the intervention was delivered, feelings of connection with other carers were evident among the participants. This was mainly achieved by the participants becoming aware that their emotional experiences were normal and common among other carers through the intervention. This sense of connectedness is particularly important considering the high prevalence of loneliness in family carers of people with dementia (i.e., 61.4% of carers reported feeling at least moderate loneliness; Victor et al., 2021).

Previous studies have found that family carers of people with dementia desire interpersonal support (Contreras, Mioshi et al., 2021; Lauritzen et al., 2015). Although opportunities for face-to-face interpersonal communication with the therapist were limited due to the nature of the intervention, the findings here highlight that participants still benefitted from the written feedback in this study. Notably, feelings of loneliness were likely aggravated during the COVID-19 pandemic as a consequence of the public health restrictions (Alzheimer’s Society, 2020). In this context, a sense of connection with other participants, and the positive therapeutic relationship with the therapists, appeared central to the acceptability of the intervention. This feeling was also shared by the therapists that took part in the feasibility study (Contreras, Van Hout et al., 2021).

A combination of several factors made the online mode of delivery acceptable for these carers. The online delivery increased flexibility around completion of sessions. The platform in which the intervention was delivered was easy to access and navigate and the time and effort required to complete the intervention was perceived as feasible. Previous studies have reported several advantages in using internet-delivered interventions with family carers of people with dementia. For instance, internet-based interventions facilitate access to support among family carers who are often unable to leave the care recipient unattended, as well as those limited by geographic and mobility constraints (Lappalainen et al., 2021).

Despite such advantages and the fact that online interventions may be highly acceptable to service users, some healthcare professionals may be hesitant regarding the implementation of this type of technology-based intervention. Some healthcare professionals still believe that online-delivered interventions are less effective than face-to-face interventions due to the lack of personal contact and the extra challenges to build a strong therapeutic relationship (Kuso et al., 2021; Sucala et al., 2012; Thew, 2020; Wehmann et al., 2020). As a consequence, these professionals may decide not to signpost carers to these types of online interventions. Future research should aim to address these concerns among healthcare professionals to achieve successful implementation of online interventions for family carers of people with dementia.

Overall, the intervention was acceptable to the participants, but some challenges were also identified. One of the key areas for improvement identified was enhancing the interaction between participants and therapist. The carers expressed a need for some face-to-face and bi-directional interactions, such as having the opportunity to have a video call with their assigned therapist. A recent systematic review on blending face-to-face and internet-based interventions demonstrated that the blended approach can save clinician time, produce lower dropout rates and are cost-effective (Erbe et al., 2017). This additional face-to-face element, even if it is virtual, may lead to increased benefits, and thus should be considered in a full-scale effectiveness trial.

Other potential challenges included limited flexibility of some programme functions. Some carers had concerns about not remembering the responses they had made to questionnaires in previous sessions as they did not have access to these records once the session had been completed. In addition, since the participants had a set time frame (i.e., 12 weeks) to complete all eight sessions, they wished they had more flexibility in when to start the next session. Providing additional aide-memoire and more flexible scheduling of sessions may need to be considered in a full-scale trial to enhance the participants’ experience of the intervention.

Despite the increasing number of online interventions developed in recent years, studies assessing the acceptability of those interventions remain scarce. A recent systematic review on usability and acceptability of internet-based interventions for family carers of people with dementia found that only three out of ten studies used qualitative interviews to explore carers’ in-depth views on acceptability (Ottaviani et al., 2021). None of those three interventions focused solely on psychological components; instead, their main focus was the provision of an online social support platform or education. Furthermore, all three interventions differed in delivery methods, including two that did not involve therapists (Dam et al., 2019; Duggleby et al., 2019; Kovaleva et al., 2019). The current study provides evidence supporting that internet-delivered guided self-help psychotherapy, which includes interactions with an online therapist, is acceptable to dementia family carers presenting at clinical levels of depression and anxiety at baseline.

There were some limitations to the study. This intervention was tested in a group of carers who did have internet access. Moreover, no data was collected on how much knowledge and confidence participants had about using the internet and how often they used the internet at baseline. Therefore, it is possible that those with little or no computer literate declined to take part in the study. Most of the participants who took part in the feasibility study were wives and daughters of the person with dementia (i.e., female carers). Thus, the experiences of male carers are underrepresented. Future research should explore the acceptability of this type of intervention and the reasons for the lower uptake among male carers. The information on the ethnicity of carers was not collected as part of the study. However, the participants were mainly recruited from the East of England and Greater London areas, where most of the community is White British, and so the results may not be generalizable to a broader population. The acceptability of iACT4CARERS should be explored among carers from ethnic minority groups to enable investigation of wider implementation.

### Conclusion

Overall, internet-delivered, guided, self-help ACT was acceptable to family carers of people with dementia who were able to access it. Positive experiences and the acceptability of the intervention were facilitated by learning helpful ACT skills, perceiving the content of the session relatable to the carers’ needs, feeling connected to other carers and the therapist during the study, noticing the benefits of the intervention and the user-friendliness of the online platform. It is recommended that a future full-scale effectiveness trial includes some “face-to-face” bidirectional interactions, such as video calls, between participants and therapists. Improving the flexibility of some of the programme functions (such as the ability to rehearse past learning and more flexible “on-demand” access to session content) may further improve acceptability of the intervention to participants in a future trial. These suggested improvements should also be considered in the development of other future internet-delivered, guided self-help psychotherapies for family carers of people with dementia.

## Supplementary Material

Supplemental Material

## Data Availability

The data are not publicly available due to their containing information that could compromise the privacy of research participants.
