# Supplementary material for: Internet-delivered guided self-help Acceptance and Commitment Therapy for family carers of people with dementia (iACT4CARERS): a qualitative study of carer views and acceptability
Source: Int J Qual Stud Health Well-being. 2022 Apr 17;17(1):2066255. doi: 10.1080/17482631.2022.2066255 (PMC9037213; doi:10.1080/17482631.2022.2066255)
Supplement: Supplemental Material [file ZQHW_A_2066255_SM9147.zip › Supplementary/Supplementary material 2.docx]

**Participant Interview**

Thank you very much for taking part in the study and also agreeing to take part in the interview.

I will be audio recording the interview using a digital voice recorder. This is to make sure that I don't miss anything you say and to help us summarise the results of the interviews.

Is it okay to turn on the digital voice recorder now? [Turn the audio recorder on]

1. First, I am interested in hearing your general opinions of online Acceptance and Commitment Therapy. Before I ask you any questions that might get you thinking about specific things, would you please give me your general opinions of this online programme?

**The acceptability and relevance of online ACT (e.g., appropriateness, user-friendliness of online ACT)**

1. Can you please tell me the main things you learned in the online programme?
2. Did those things you learned have good fit with your current needs or what you value?

- If yes, could you please try to tell me how things you learned fitted with your current needs (or what you value)?
- If not, could you please try to tell me how things you learned did not fit with your current needs (or what you value)?

1. How do you feel about the user-friendliness of the online programme? For example, did you find easy to log in and then navigate through the programme?
2. How did you find the feedback from your therapist? Were there any helpful or unhelpful aspects of the feedback?

**The feasibility and burden (e.g., time, frequency, location and format of face-to-face sessions)**

1. How do you feel about the amount of time and effort required to participate in the online programme?
2. Did anything stop you from completing the programme?

**Perceived benefit (e.g., reduced psychological distress)**

1. I am interested in whether you got anything out of the online programme. Did you benefit in any way?

- If yes, could you please try to tell me how the online programme had this good effect?

1. Do you think the online programme had any bad effects?

- If yes, could you please try to tell me how the online programme had a bad effect?

1. The online programme was meant to support you in unhooking yourself from internal struggles and taking meaningful actions towards what matters to you in your life. Do you think the online programme did anything to help you take meaningful actions towards what you value?

- If yes, can you tell me some examples of actions you have taken towards what you value?

Group attendees only

1. Do you think the optional peer support group sessions had a good effect?

- If yes, could you please try to tell me how the optional group sessions had a good effect?
- If not, could you please try to tell me in what way it was not helpful?

Group non attendees only

1. Did you want to attend the optional peer support group sessions? If yes, what were the main barriers for attending groups?

**Adaptation (e.g., suggestions for improvement)**

1. Do you have any suggestions for improvement for the online programme?
